# Analyzing the factors affecting virus invasion by quantitative single-particle analysis

**DOI:** 10.1080/21505594.2024.2367671

**Published:** 2024-06-23

**Authors:** Yi-Ning Hou, Li-Juan Zhang, Lei Du, Dan-Dan Fu, Jing Li, Liu Liu, Peng-Fei Xu, Ya-Wen Zheng, Dai-Wen Pang, Hong-Wu Tang

**Affiliations:** a College of Chemistry and Molecular Sciences, Wuhan University, Wuhan, China; b College of Chemistry, Nankai University, Tianjin, China

**Keywords:** Virus invasion, quantitative single-particle analysis, Semliki forest virus, Japanese encephalitis virus, influenza a virus

## Abstract

Viral diseases are among the main threats to public health. Understanding the factors affecting viral invasion is important for antiviral research. Until now, it was known that most viruses have very low plaque-forming unit (PFU)-to-particle ratios. However, further investigation is required to determine the underlying factors. Here, using quantitative single-particle analysis methods, the invasion of Semliki Forest virus (SFV), Japanese encephalitis virus (JEV), and influenza A virus (IAV) containing attachment to the cell surface, entry into the cell, transport towards the cell interior, and fusion with endosomes to release nucleocapsids were quantitatively analysed in parallel. It was found that for SFV with an PFU-to-particle ratio of approximately 1:2, an entry efficiency of approximately 31% limited infection. For JEV, whose PFU-to-particle ratio was approximately 1:310, an attachment efficiency of approximately 27% and an entry efficiency of 10% were the main factors limiting its infection. Meanwhile, for IAV with PFU-to-particle ratios of 1:8100, 5% attachment efficiency, 9% entry efficiency, and 53% fusion efficiency significantly limited its infection. These results suggest that viruses with different infectivities have different limited steps in the invasion process. Moreover, there are significant differences in attachment efficiencies among viruses, emphasizing the pivotal role of attachment in viral invasion. The influence of the virus purification method on virus invasion was also investigated. This study, for the first time, reports the efficiencies of different stages of virus invasion, leading to a better understanding of virus invasion and providing a protocol to quantitatively analyse the virus invasion efficiency.

In the process of viral infection, we could not only know how viruses infect cells, but a profound and accurate understanding of the efficiency of infection is equally crucial. By quantitatively analysing the virus infection process and measuring how many viruses can succeed in each step, we can gain a more comprehensive understanding of viral infection, which has significant implications for the therapy of viral diseases, research on vaccines, and the use of viral vectors [1,2]. According to the measurement of the PFU-to-particle ratio, which is a fundamental tool in virology for assessing the infection of virus particles, it has become evident that a significant number of potentially infectious virions fail to infect under standard experimental conditions; however, the influencing factors remain elusive [3,4].

The process of viral infection is intricate, starting with the attachment of the virus to the cell surface via auxiliary attachment factors and specific receptors. Subsequently, internalization occurs through endocytosis or micropinocytosis. The virus then undergoes trafficking facilitated by actin and/or microtubules, fusing with the membrane of endosomes [5,6]. After virus uncoating, the viral ribonucleoprotein complexes (vRNPs) are released separately into the cytosol, and then transported to the nucleus or other specific sites inside the cell for replication and expression [7]. Subsequently, the new viral particles were assembled and released. Many viral infection attempts turn out to be futile, leading to significant changes in PFU-to-particle values. For virus attachment, it was found that the constrgaints of infection seem to be limited to only a small fraction of viral particles actually encountering cells for HIV-1 [8]. Moreover, certain HIV particles fail to attach to the cell membrane because of the lack of gp120 on their surfaces [9]. In addition, most influenza viruses are unable to enter the cell because of the short-lived and abortive recruitment of clathrin and dynamin [10]. For membrane fusion, it was reported that among the dengue virus particles that bind to the cell surface, only 17% ultimately undergo membrane fusion [3]. It was also found that the interferon-induced transmembrane protein (IFITM) family, located on cytoplasmic and endosomal membranes, can hinder the membrane fusion of various viruses, including influenza A virus and dengue virus [11]. Quantitative confocal microscopy revealed that the WSN strain of IAV exhibited 65–70% membrane fusion efficiency and 80% genome release efficiency [12]. These results suggest that each stage of virus invasion impacts virus infection. However, it remains unclear how these aborted processes influence the infection of viruses, whether viruses with different infectivities have the same limited step in invasion, and how different in their efficiency in each invasion step.

In this study, quantitative single-particle analysis methods were employed to precisely observe the invasion of three distinct infective viruses in parallel, and to compare the efficiency of different stages of virus invasion, including attachment, entry, intracellular transport, and membrane fusion steps. Using a lipid-specific method, we used the virus without ultracentrifugation or ultrafiltration purification, excluding the effect of purification on viral infection. This finding revealed that there was notable variation in the infection limitations among different infectious viruses. For the Semliki Forest virus (SFV), with an PFU-to-particle ratio of approximately 1:2, the 31% entry efficiency limits its infection. For Japanese encephalitis virus (JEV) with an PFU-to-particle ratio of 1:310, both 27% attachment efficiency and 10% entry efficiency were identified as major obstacles. For the influenza A virus (IAV) with an PFU-to-particle ratio of 1:8100, limitations were observed in the form of 5% attachment efficiency, 9% entry efficiency, and a significant 53% fusion efficiency. These results suggest that different viruses have different limiting steps in viral infection. Additionally, our findings revealed that inefficient binding substantially varied the virus infectivity; the maximum attachment efficiency of SFV was approximately 96%, while that of JEV and IAV were only 27% and 5%, respectively. Furthermore, the effects of ultracentrifugation and ultrafiltration purification on viral invasion were analysed. By employing a protocol to quantitatively analyse the efficiency of virus stages during invasion, we obtained a more accurate understanding of virus infection.

## Materials and methods

### Cell culture

Baby hamster Syrian kidney 21 (BHK-21) and African green monkey kidney (Vero) cells were cultured in Dulbecco’s modified Eagle’s medium (DMEM) (Gibco^TM^) supplemented with 10% foetal bovine serum (FBS) and 100 U/ml penicillin-streptomycin. Madin-Darby canine kidney (MDCK) cells were cultured in DMEM supplemented with 5% FBS and 100 U/ml antibiotics. All cells were maintained in an atmosphere of 5% CO_2_ at 37°C. To ensure a consistent physiological state of the cells, mycoplasma contamination was assessed after revival. Experiments began two days after cell revival. The cells were discarded before 20 divisions. For fluorescence imaging, cells were cultured in 20 mm glass-bottomed dishes. BHK-21 cells were utilized for propagating JEV. Vero cells were employed for all infection experiments involving SFV and JEV, except for SFV propagation. MDCK cells were utilized for both infection experiments and propagation of IAV.

### Virus propagation and purification

JEV strain SA-14-14-2 was propagated in BHK-21 cells. IAV strain A/PR/8/34 (H1N1) was propagated in MDCK cells. The SFV strain SFV4 was kindly provided by Dr. Xi Zhou from the Wuhan Institute of Virology and propagated in Vero cells. After 2 days of virus infection, viruses were collected exclusively from cell supernatants without cell lysis, clarified by centrifugation at 1,500 × g for 10 min to eliminate cell debris. Then the virus was aliquoted and stored at −80°C.

To analyse the effect of purification on viral infection, virus was divided into three parts. One part was left untreated and used as the control group. Another part was purified using ultrafiltration centrifugal tubes (Millipore, MWCO 100-kDa) at 2000 × g and 4°C for 20 min. The remaining part was purified by ultracentrifugation. For SFV and JEV, the virus was firstly concentrated by centrifugation at 125,000 × *g* and 4°C in a Ty45 Ti rotor (Beckman) for 120 min. The resulting pellet was resuspended in PBS and then centrifuged on 10–35% potassium tartrate-glycerol (30%) at 125,000 × *g* in an SW32 Ti rotor for 120 min. Finally, the virus was desalted at 180,000 × *g* for 60 minutes [13]. For IAV, the progeny viruses were harvested from cell supernatants through ultracentrifugation at 110,000 × g for 90 minutes using a Ty45 Ti rotor (Beckman). Subsequently, the viruses underwent fractionation on a 15%−60% (w/v) discontinuous sucrose gradient and were centrifuged at 110,000 × g for 1 hour in a Beckman SW28 rotor. The resulting viral bands were collected and removed sucrose from the viral suspension at 120,000 × *g* for 60 minutes [10].

### Virus infectivity analysis

The PFU of viruses was determined using a plaque assay [13]. The number of viral genome-containing particles (GCPs) was determined using quantitative PCR, as previously described [14]. Virus RNA was extracted using TRIzol reagent (Invitrogen). Following the manufacturer’s instructions, RNA samples were amplified using RNA-Direct Real-Time PCR Master Mix (Toyobo). Post amplification, DNA melting curve analysis was performed to determine the specificity of the PCR products. The standard curve of infectious clone plasmids was used to quantify the genome RNA copy number. A plasmid containing a fragment of the IAV genome was constructed in our lab [15]. Plasmids containing fragments of JEV and SFV genomes were provided by Dr. Gengfu Xiao from the Wuhan Institute of Virology and were purchased from Addgene, respectively. The qPCR primers targeting the SFV nsP1 gene were 5′- ACAGACTGTCACTGAGCAG-3′ and 5′- GTGACCATCTACTGCAGAGA-3′ [16]. The qPCR primers targeting the JEV NS5 gene were 5′-AGCTTCTAGATGGTGAACACCGCA-3′ and 5′-TCACGTCCATCACGGTCTTTCCTT-3′. The qPCR primers targeting the IAV M1 gene were 5′- ATGAGTCTTCTAACCGAGGTCG-3′ and 5′- TGCAAAGACATCTTCAAGTCTCTG-3′. The physical particles of the viruses were quantified using a ZetaView nanoparticle-tracking analyser.

### Virus labelling

To fluorescently label the virus, a biotin-streptavidin (SA)-based lipid-specific method was used [13]. Briefly, viruses were incubated with 30 μM DSPE-PEG (2000)-biotin (Avanti) at room temperature for 1.5 h. Unincorporated biotin was removed using NAP-5 gel filtration columns (GE Healthcare). Virus aggregates were eliminated with 0.22 μm-pore-size filters (Millipore). To determine the number of virus particles bound to the cell surface, Biotinylated viruses were incubated with cells at 4°C for indicated durations. 2 nM SA-QD705 (Wuhan Jiayuan) were subsequently added to the cells to conjugate with the biotinylated viruses at 4°C. Unbound viruses and QDs were washed with pre-chilled PBS. To quantitatively analyse virus endocytosis, the cells with QD-labelled viruses on the surface were immediately warmed to 37°C to initiate viral infection. After indicated durations, the cells were transferred to 4°C to terminate the virus infection and to allow the virus outside the cells labelled with 2 nM SA-Cy3 (Thermo). After fixation with 4% paraformaldehyde at 4°C for 10 min and then at room temperature for 30 min, the cells were imaged under a spinning-disk confocal microscope. To analyse the membrane fusion event, viruses were stained with 0.2 μM DiO and 0.4 μM R18 (Millipore) at room temperature for 60 min as reported [17].

To incubate the cells with different numbers of viruses, a certain number of cells was inoculated into the dish. The virus stocks with known concentrations were then diluted with different volumes of PBS buffer. To ensure that all cells in the dish could be fully incubated with the virus particles, 300 μL of virus solution was added to the dish. The total number of virus particles in the 300 μL volume divided by the number of cells in the dish was the number of virus particles incubated with each cell.

### Labeling microtubule and clathrin of cell

Transfection of cells with plasmids expressing GFP-microtubule-associated protein 4 (GFP-MAP4) to label the microtubules and plasmids expressing EGFP-clathrin light chain (EGFP-Clc) to label the clathrin. Cells were cultured in a 24-well plate, and after 12 hours, 0.5 μg of DNA was transfected into the cells using Lipofectamine LTX reagent (Life Technologies) following the manufacturer’s instructions. After incubating at 37°C for 24 hours, cells were transferred to 20 mm glass-bottomed dishes for fluorescence imaging.

### Flow cytometry

Cells were initially cultured in 6-well plates for 12 hours before virus infection. Following this, the cells underwent infection with different labelled viruses for varying durations as dictated by the experimental design. Upon completion of the infection period, the cells were gently detached from the culture plates using trypsin. After remove trypsin, the cell was resuspended in 4% paraformaldehyde and allowed to incubate at room temperature for 15 minutes to ensure proper fixation. Following fixation, the cells was then resuspended in PBS and vigorously vortexed. After filtration to eliminate any remaining clumps or debris, the prepared cell suspension was subsequently subjected to analysis using the FACSAria III flow cytometer (BD Biosciences). Flow cytometer parameters were meticulously configured in accordance with the specific requirements of the experiment, and data acquisition proceeded accordingly.

### TEM imaging

Five microlitres of fresh viral suspension were dropped onto carbon-coated copper grids, and the excess viral suspension was removed using filter paper. The grids were then stained with 3 μL 2% (w/v) phosphotungstic acid for 3 min and dried overnight. TEM imaging was performed using a JEOL-JEM2100 transmission electron microscope (TEM).

### Fluorescence imaging

Fluorescence images were acquired using a spinning-disk confocal microscope (Revolution XD; Andor) equipped with an EMCCD (Andor iXon Ultra897). DPSS lasers at 488, 561, and 640-nm and Chroma emission filters of 525/50, 605/20, and 685/40-nm were used for DiO, Syto82/Cy3/R18, and QD705 imaging, respectively. To quantitatively analyse virus infection, a *z*-step of 0.3 μm was used for 3D imaging of cells.

### Image analysis

Line profiles showing distributions of the QD and Syto signals were acquired with Image-Pro-Plus. 3D reconstruction of cells was obtained using Vaa3D software. Import image files containing the cell surface virus binding information into the Vaa3D software. Use 3D viewer for entire image to perform 3D reconstruction of the three-dimensional structure of cells and the distribution of viruses. Adjust the viewing angle and parameters, and then save the projection images. The number of viruses were obtained from 3D reconstruction images by identifying and counting the fluorescent spots representing viruses with ImageJ software. Utilize tools within the Image J to select appropriate thresholds and parameters to accurately identify viruses, and use point counting and object analysis tools to quantify the number of viruses. The fluorescence intensity of viruses was obtained from raw images with ImageJ software by identifying representing viruses and aligning corresponding cellular endocytic structures in each frame. Regions of interest (ROIs) were used to perform the analysis. Image-Pro-Plus software (Media Cybernetics) was used to align the coordinates and intensities of the points representing the virus in each frame, and to reconstruct integral trajectories of virus within the focal plane from the original image. The speed and displacement of trajectories were also obtained from Image-Pro-Plus. Cell flow cytometry data were analysed by Flow Jo software.

### Statistical analysis

Virus attachment efficiency was calculated as the ratio of the number of viruses bound to the cell surface to the total number of viruses added. The entry efficiency was determined as the ratio of the number of viruses that successfully entered the cells to the number of viruses bound to the cell surface. Transport efficiency referred to the proportion of internalized viruses that underwent microtubule-dependent transport behaviour. Data are expressed as means ± standard deviation (SD). Student’s t-tests were conducted using the original, non-normalized dataset for all statistical analyses.

## Results

### Mildly and efficiently labelling viruses with quantum dots

**Figure 1.**
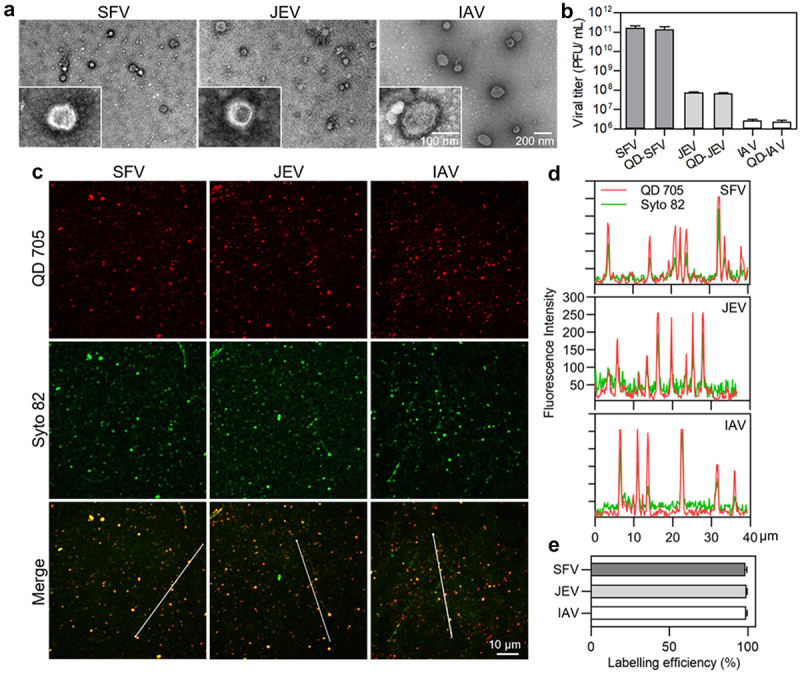
Mildly and efficiently labelling viruses with QDs. (a) TEM images of SFV, JEV and IAV particles. (b) Infectious titers of viruses and QD-labeled viruses. (c) Fluorescence images of viruses labeled by QD 705 and stained with Syto 82 nucleic acid dye. (d) Line profiles showing distributions of the QD and Syto signals on the lines in C. (e) The efficiencies of QDs labelling the Syto-stained viruses.

SFV, JEV, and IAV are enveloped viruses (Figure 1a) that infect host cells mainly via the endocytic pathway [10,18,19]. However, their infectivities vary significantly. The PFU-to-physical particle ratios of SFV, JEV, and IAV were 1:2, 1:310, and 1:8100, respectively, whereas the PFU-to-GCP ratios of the viruses were 1:1.3, 1:122, and 1:3900, respectively (Table 1). This result suggested that the absence of genomes of some virus particles was one of the causes for the low infectivity of virus populations, but not the main cause, especially for IAV. To further explore the factors affecting viral invasion, the invasion processes of SFV, JEV, and IAV were quantitatively analysed in parallel, including virus attachment to cell surfaces, entry into the cells, transport towards the cell interior region, and membrane fusion for nucleocapsid release. Vero is the most commonly used model cell in studies of SFV and JEV invasions. MDCK cells are the most commonly used model cells in IAV studies. Hence, invasion with SFV and JEV in Vero cells and IAV invasion in MDCK cells were investigated in this study.

**Table 1.** The infectivity of SFV, JEV and IAV (*n* = 3).

Viruses	PFUs/ml	GCPs/ml	Particles/ml	PFU:GCP ratio	PFU:particle ratio
SFV	(2.1 ± 0.4) × 10^11^	(2.8 ± 0.5) × 10^11^	(4.2 ± 0.4) × 10^11^	1: 1.3	1: 2
JEV	(6.9 ± 0.2) ×10^7^	(1.4 ± 0.3) × 10^10^	(3.6 ± 0.2) × 10^10^	1: 122	1: 310
IAV	(3.3 ± 0.5) × 10^6^	(1.3 ± 0.2) × 10^10^	(2.7 ± 0.1) × 10^10^	1: 3900	1: 8100

### Quantitatively analyzing the attachment efficiency

**Figure 2.**
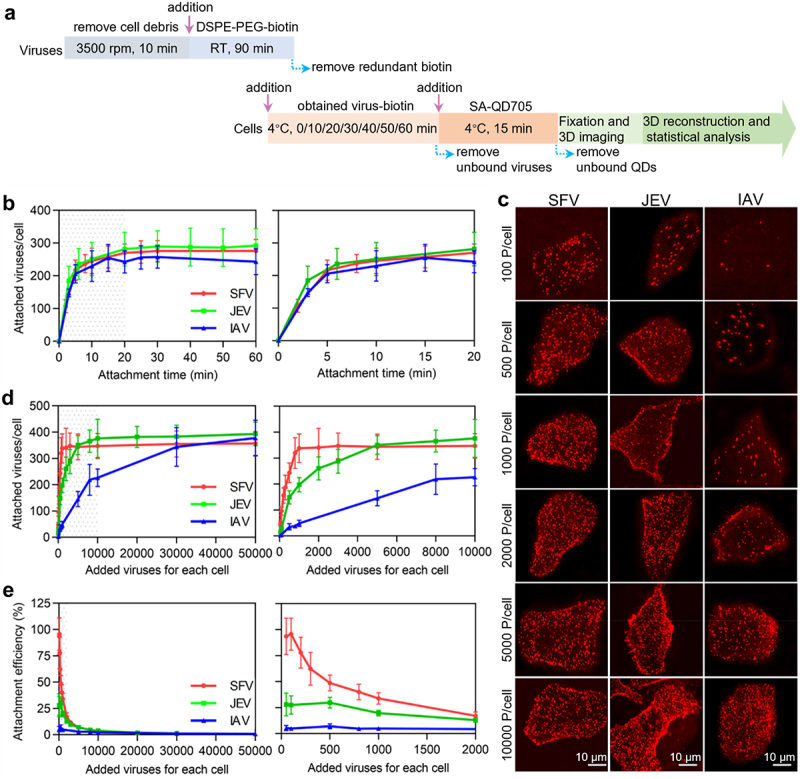
Quantitatively analyzing the efficiency of virus attachment to cells. (a) Flow chart for quantitative analysis of virus attachment. (b) SFV, JEV and IAV particles were incubated with cells at 4°C for 0, 10, 20, 30, 40, 50 and 60 min. After fixation, the cells were imaged in 3D and the number of viruses attached to the cell surface were counted with ImageJ (n = 50). (c) Different amounts of virus particles were added to cells, allowed to be attached to cells, labeled with QDs, and then imaged in 3D. The panels are the z-projection images of cells attached with SFV, JEV and IAV, respectively. (d) Different amounts of viruses were added to cells. The cells were imaged in 3D and the number of viruses attached to cells was quantified (n = 50). (e) The efficiencies of virus attachment to cells (n = 50). In b, d and e, the right panels are the enlarged views of the shadow region of the left panels.

Binding to host cells is the first step in infection. To analyze the attachment efficiencies of the viruses to cell surface, a certain number of viruses were incubated with a certain number of cells at 4˚C, allowing viruses to be attached to the cell surface. After labelling the viruses with QDs and fixation, the cells were imaged in three dimensions (3D) under a confocal microscope at *z*-intervals of 0.3 μm. The number of viruses in each cell was determined by quantifying the QD spots using ImageJ software (Figure 2a). Analysis of the kinetics of virus attachment revealed that the number of viruses attached to the cells sharply increased in the first 5 min and gradually reached a plateau in the next 15 min (Figure 2b). The SFV, JEV, and IAV followed nearly the same attachment kinetics. Hence, in subsequent experiments, the viruses were incubated with the cells for 20 min for full attachment.

To analyse the virus attachment efficiency, various numbers of viruses in a certain volume were incubated with the cells. It could be visually observed that the number of viruses attached to a cell increased with the number of viruses incubated with the cell (Figure 2c). However, when the same number of viruses was incubated with the cells, such as 100, 500, or 1000 virus particles per cell, the amount of SFV bound to the cell was obviously higher than that of JEV, which was more than that of IAV. Statistics revealed that when the number of viruses incubated with cells increased, SFV particles first reached attachment saturation on the cell surface and IAV particles finally reached attachment saturation (Figure 2d). Specifically, for SFV, the number of viruses bound to a cell rapidly increased as the number of viruses incubated with the cell increased from 50 to 1000 particles per cell (Figure 2d, red curve). When 1000 or more viruses were incubated with the cell, the number of viruses bound to the cell was approximately 340. For JEV, the number of viruses bound to a cell increased as the number of viruses incubated with the cell increased from 50 to 10,000 particles per cell (Figure 2d, green curve). When 10,000 or more viruses were incubated with the cells, the number of viruses bound to the cells was approximately 380. For IAV, the number of viruses bound to the cell slowly increased until the virus incubated with the cell reaching 50,000 particles per cell (Figure 2d, blue curve). At this point, the number of IAV particles bound to each cell reached approximately 380.

When 50–100 viruses were incubated with each cell, the attachment efficiency was the highest (Figure 2e). The maximum attachment efficiencies of the SFV, JEV, and IAV were approximately 96% (96/100), 27% (27/100), and 5% (5/100), respectively. When 1000 SFV 10,000 JEV or 50,000 IAV particles were incubated with cells, the viruses bound to the cells were nearly saturated. At this point, the attachment efficiencies of the viruses were approximately 34% (340/1000), 3.8% (380/10000), and 0.4% (380/100000). In other words, the more virus particles incubated with the cell, the lower the proportion of viruses attached to the cell surface.

Vero and MDCK cells were similar in size (Figure S1). SFV, JEV, and IAV were nearly spherical particles of approximately 45–75 nm, 45–65 nm and 70–190 nm, respectively (Figure 1a and Figure S2). The maximum number of SFV bound to a Vero cell (340), JEV bound to a Vero cell (380), and IAV bound to an MDCK cell (380) suggested that the size of the virus particles was not the key factor affecting virus attachment. There were probably more IAV receptors on MDCK cells and JEV receptors on Vero cells than SFV receptors on Vero cells. According to structural biology research, there are 80 trimeric envelope glycoprotein spikes on each SFV particle, 90 dimeric envelope glycoprotein spikes on each JEV particle, and 300–400 trimeric envelope glycoprotein spikes on each IAV particle [14,20,21]. It was speculated that this might be due to the binding force of viral glycoprotein to the receptor or the number of glycoproteins with exposed receptor-binding sites that limited the attachment of JEV and IAV to the cells.

### Quantitatively analyzing virus entry efficiency

**Figure 3.**
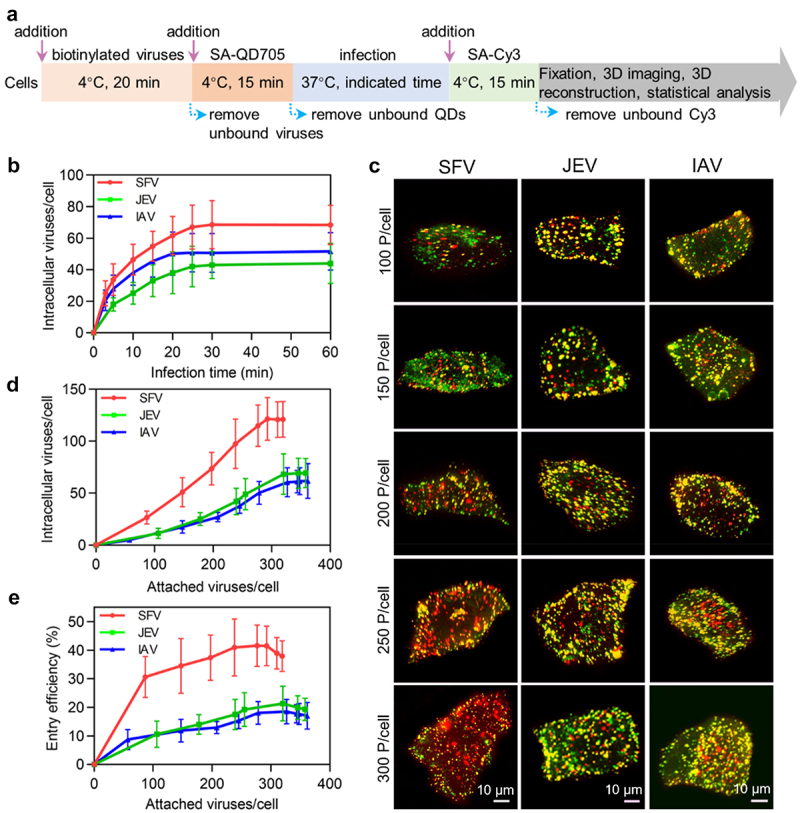
Quantitatively analyzing the efficiency of virus entry. (a) Flow chart for quantitative analysis of virus entry. (b) SFV, JEV and IAV were allowed to infect cells for 10, 20, 30, 40, 50 and 60 min. Then the cells were imaged in 3D and the number of viruses inside the cells was determined with ImageJ (n = 50). (c) Different amounts of viruses were allowed to bind to cells, labeled with QD705 (red), and allowed to infect cells for 30 min. The viruses remained on the cell surface were then stained with Cy3 (green). After that, the cells were imaged in 3D. The panels were the z-projection images of the cells. The red spots were intracellular viruses and the yellow were extracellular viruses. (d) Different amounts of viruses were allowed to be attached to cells. And the number of viruses entering cells after infection for 30 min was counted (n = 50). (e) The entry efficiencies of viruses (n = 50).

To quantitatively analyse viral entry, viruses were labelled with SA-QD705 on the surface of the cells. After infection for a certain period, viruses remaining on the cell surface were further stained with SA-Cy3 to distinguish them from the viruses internalized into the cell. Then, the cells were imaged in 3D, and the viruses labelled only by QDs in the cells were counted one by one (Figure 3a) [13]. The number of SFV and JEV particles internalized into the cell gradually increased in the first 25 min of infection and rarely changed over the next 35 min (Figure 3b, red and green curves). This result is consistent with previous reports on the entry kinetics of viruses [13,22]. Meanwhile, the number of IAV particles entering the cell plateaued after 20 min of infection (Figure 3b, blue curve). Based on this result, viruses were allowed to infect cells for 30 min to maximally enter the cells in subsequent experiments.

By altering the number of viruses incubated with cells, different numbers of viruses could bind to the cell surface. It could be visually observed that after infection for 30 min, the amount of SFV, JEV and IAV particles internalized into the cells increased with the number of viruses initially attached to the cell surface (Figure 3c). More interestingly, in the cells initially attached to nearly the same number of viruses, more SFV particles entered the cell than JEV and IAV. Statistically, when the number of SFV particles attached to the cell increased from approximately 87 to 293, the number of viruses internalized into the cell after 30 min of infection increased from approximately 27 to 122 (Figure 3d, red curve). As for JEV, when the viruses initially attached to the cell increased from 106 to 346, the number of viruses entering the cell increased from approximately 11 to 69 (Figure 3d, green curve). Meanwhile, when the IAV particles initially attached to the cell increased from approximately 57 to 327, the number of viruses entering the cell increased from approximately 5 to 61 (Figure 3d, blue curve). By further increasing the number of viruses attached to the cell until saturation, the number of viruses entering the cell did not continue to increase.

Correspondingly, the entry efficiencies of the SFV, JEV, and IAV particles ranged from approximately 31% (27/87) to 42% (122/293), 10% (11/106) to 20% (69/346), and 9% (5/57) to 19% (61/327), respectively (Figure 3e). The more virus particles attached to the cell surface, the higher the proportion of viruses internalized into the cells, which was in contrast to the case of virus attachment. These results were consistent with previous reports that concentration stress caused cells to take up extra extracellular material, but there was a maximum limit [23].

### Quantitatively analyzing virus transport efficiency

**Figure 4.**
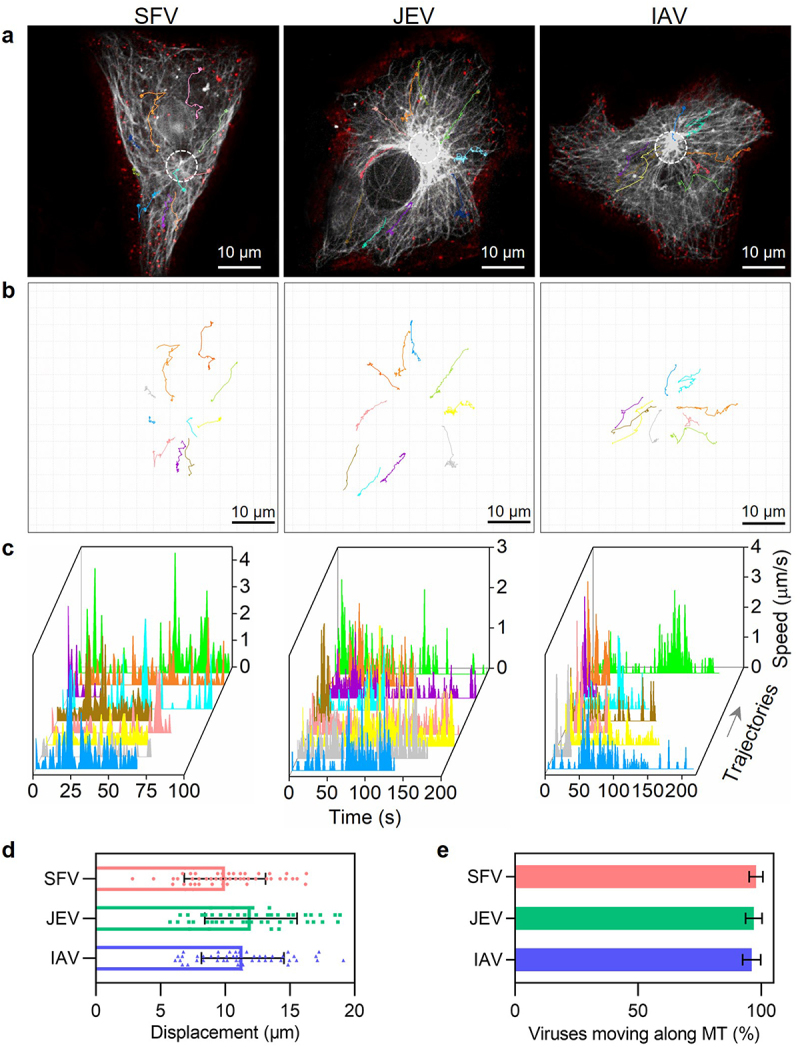
Quantitatively analyzing the efficiency of virus transport toward the cell interior. (a and b) trajectories of QD705-labeled viruses (red) moving in cells with GFP-labeled microtubules (gray). The white circles in a indicate the MTOC of the cells. (c) The speed vs time plots of the trajectories. (d) Displacements of viruses moving along microtubules toward the MTOC (n = 50). (e) The proportions of viruses moving along microtubules toward the MTOC.

After entering the cells through endocytosis, SFV, JEV, and IAV are transported by the microtubule-based transport system towards the cell interior region [6,13,24]. Because the transport of different virus particles is nonsynchronous and might be full of twists and turns [6], the transport efficiencies of SFV, JEV, and IAV particles were quantitatively analysed by real-time single-virus tracking. By monitoring the invasion of QD705-labelled viruses in cells with GFP-labelled microtubules, it was observed that SFV, JEV, and IAV all moved along the microtubules towards the cell interior region after entering the cells (Figure 4a). The trajectories of the viruses were winding, but generally towards the microtubule organizing centre (MTOC) of the cells (Figure 4b). Speeds of the viruses were sometimes fast to several micrometres per second and sometimes very slow (Figure 4c). The displacements of the viruses ranged from several micrometres to tens of micrometres (Figure 4d). These results are consistent with our previous reports on the dynamic behaviour of microtubule-dependent movements [25]. The virus transport efficiency within cells can be measured by calculating the ratio of the number of viral particles transported along microtubules to the total number of viruses that entered the cell. By incubating 500 SFV, 2000 JEV, and 10,000 IAV particles with the cells and randomly analysing the intracellular transport behaviours of the viruses, it was found that during 30 min of infection, approximately 97% (97/100) of internalized SFV, JEV, and IAV particles were transported along MTs from the cell edge towards the cell interior region (Figure 4e). Further studies suggested that the transport efficiency of viruses did not change with the number of viruses incubated with the cells or internalized into the cells (Figure S3(a)). However, it cannot be ruled out that the remaining viruses were transported into the cell interior 30 min after infection.

### Quantitatively analyzing virus membrane fusion efficiency

**Figure 5.**
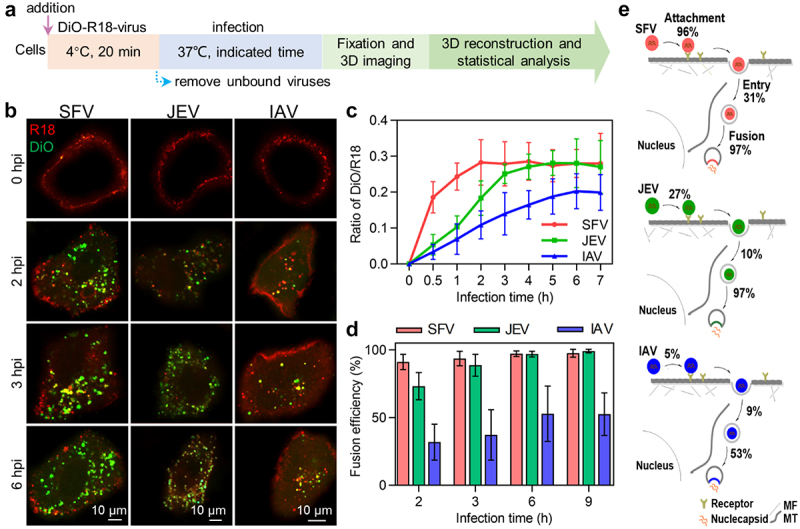
Quantitatively analyzing the efficiency of virus membrane fusion. (a) Flow chart for quantitative analysis of virus membrane fusion. (b) DiO-R18-labeled viruses were allowed to infect cells for different time periods. (c) DiO fluorescence intensity-to-R18 fluorescence intensity ratios in cells infected by DiO-R18-labeled viruses measured by flow cytometry (n = 3). (d) The membrane fusion efficiencies of viruses in cells infected by DiO-R18-labeled viruses (n = 30). (e) The efficiencies of the viruses’ different invasion stages when the viruses infect cells at low concentration.

After the viruses reach the interior region of cells, their lipid envelope fuses with the membrane of acidic endosomes carrying the viruses, releasing the nucleocapsids into the cytosol [26]. A dual-wavelength imaging method was used to analyse virus membrane fusion [17]. The viruses were simultaneously labelled with lipophilic dyes, DiO and R18, respectively, at certain concentrations (Figure 5a). Initially, DIO (green) fluorescence was suppressed due to self-quenching and fluorescence resonance energy transfer (FRET) from DIO to R18, resulting in only R18 (red) fluorescence on the virus. After the virus fused with the endosome, the DIO fluorescence increased, giving rise to the appearance of orange, yellow, and green spots, which could be distinguished from the red spots indicating failed membrane fusion (Figure 5b). The fusion efficiencies of the viruses were determined by quantifying the DiO and R18 spots in each cell.

It could be visually observed that in cells infected with DiO-R18-labelled viruses, the fluorescence signal of DiO occurred and gradually increased in number as the infection time increased (Figure 5b). Flow cytometry analysis revealed that in SFV-infected cells, the ratio of DiO fluorescence intensity to R18 fluorescence intensity rapidly increased in the first 2 h after infection and then plateaued (Figure 5c, red curve). In JEV-infected cells, the ratio slowly increased and gradually reached a plateau after infection for 3 h (Figure 5c, green curve). Meanwhile, the ratio of DiO/R18 increased very slowly and reached a plateau after 6 h of infection (Figure 5c), blue curve). This result suggests that most of the SFV, JEV, and IAV particles fused with endosomes after infection for 2, 3, and 6 h, respectively. By counting the DiO spots and R18 spots in each cell, it was found that when 500 SFV, 2000 JEV, and 10,000 IAV particles were incubated with the cells, the proportion of SFV, JEV, and IAV particles fused with endosomes 2 h after infection was approximately 91%, 73%, and 32%, respectively (Figure 5d), consistent with previous reports on the fusion of SFV and IAV [21,27]. When the infection time was extended to 6 h, the fusion efficiencies of the viruses increased to 97%, 97%, and 53%, respectively, and plateaued. Further studies showed that the fusion efficiencies of SFV, JEV, and IAV did not change with the number of viruses incubated with the cells or internalized into the cells (Figure S3(b) and(c)).

### Quantitatively analyzing the effect of ultracentrifugation and ultrafiltration purification on virus invasion

The above results were obtained using viruses treated only with low-speed centrifugation to remove cell debris, whereas in many studies, viruses were further purified by density-gradient ultracentrifugation or ultrafiltration [28,29]. In our previous work, we found that purification by ultracentrifugation greatly decreased the PFU-to-GCP ratio of viruses [13]. The results in Table 2 show that purifying viruses by ultracentrifugation decreased the PFU-to-particle ratio of both SFV, JEV and IAV by two orders of magnitude, whereas purifying viruses by ultrafiltration affected the ratio very slightly.

**Table 2.** The infectivity of SFV, ultrafiltered SFV (UF SFV) and ultracentrifuged SFV (UC SFV); JEV, ultrafiltered JEV (UF JEV) and ultracentrifuged JEV (UC JEV); IAV, ultrafiltered IAV (UF IAV) and ultracentrifuged JEV (UC IAV) (*n* = 3).

Viruses	PFUs/ml	GCPs/ml	Particles/ml	PFU:GCP ratio	PFU:particle ratio
SFV	(2.11 ± 0.4) × 10^11^	(2.84 ± 0.5) × 10^11^	(4.2 ± 0.4) × 10^11^	1: 1.3	1: 2
UF SFV	(2.34 ± 0.6) × 10^11^	(3.12 ± 0.4) × 10^11^	(4.5 ± 0.2) × 10^11^	1: 1.3	1: 1.9
UC SFV	(3.05 ± 0.7) × 10^9^	(5.21 ± 0.3) × 10^11^	(9.6 ± 0.2) × 10^11^	1: 170	1: 315
JEV	(1.47 ± 0.2) × 10^7^	(1.45 ± 0.3) × 10^9^	(4.2 ± 0.4) × 10^9^	1: 99	1: 285
UF JEV	(2.76 ± 0.3) × 10^7^	(4.67 ± 0.6) × 10^9^	(9.1 ± 0.7) × 10^9^	1: 169	1: 329
UC JEV	(3.16 ± 0.5) × 10^5^	(8.51 ± 1.2) × 10^9^	(1.1 ± 0.2) × 10^10^	1: 26930	1: 34810
IAV	(3.31 ± 0.5) × 10^6^	(1.34 ± 0.2) × 10^10^	(2.7 ± 0.1) × 10^10^	1: 3900	1: 8100
UF IAV	(6.7 ± 0.5) × 10^6^	(3.24 ± 0.2) × 10^10^	(5.6 ± 0.5) × 10^10^	1: 4700	1: 8300
UC IAV	(7.21 ± 0.5) × 10^6^	(2.31 ± 0.2) × 10^11^	(6.8 ± 0.4) × 1012	1: 32000	1: 9.4 × 10^5^

**Figure 6.**
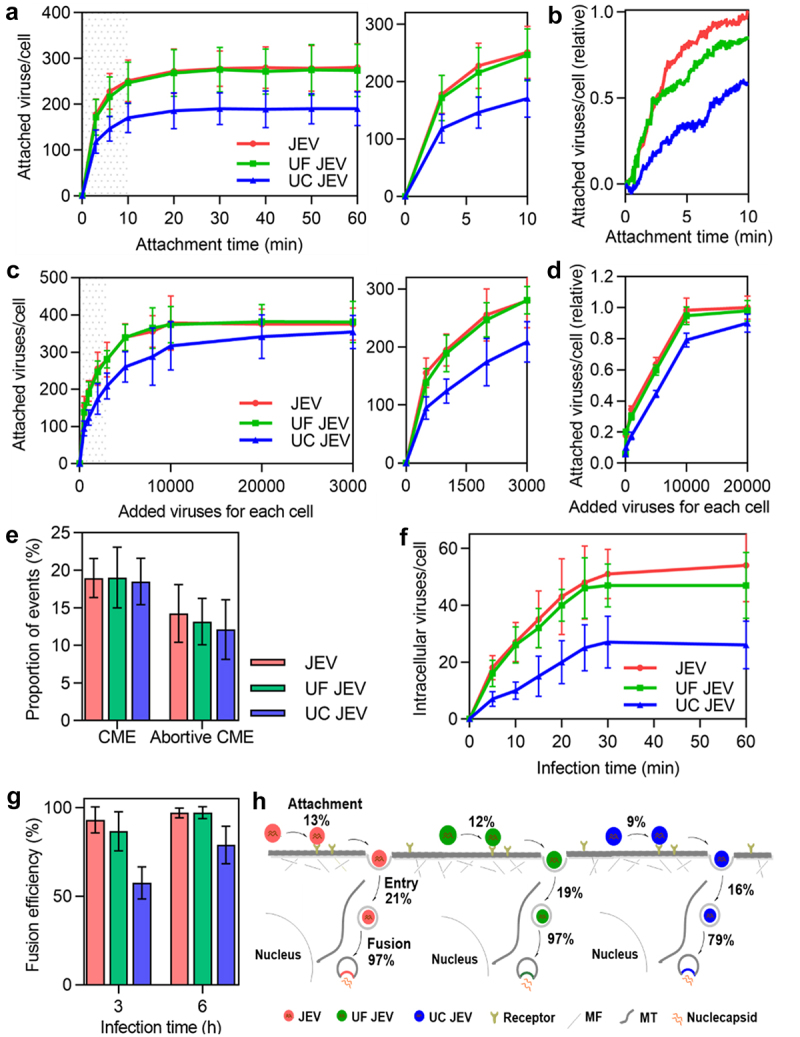
Quantitatively analyzing the effect of ultracentrifugation and ultrafiltration purification on virus invasion. (a) JEV, UF JEV and UC JEV particles were incubated with cells at 4°C for 0, 3, 6, 10, 20, 30, 40, 50 and 60 min. Then the cells were imaged in 3D and the number of viruses attached to the cell surface was determined with ImageJ (n = 50). (b) R18-labeled viruses were incubated with cells at 37°C. Its adsorption to cells in the first 10 min was monitored in real-time. (c and d) Different amounts of viruses were incubated with cells. The viruses attached to cells were quantified from 3D images (n = 50) (c) or by flow cytometry (d). (e) The proportions of viruses entering cells via CME and the proportions of viruses recruiting clathrin but failing to enter cell (n = 50). (f) JEV, UF JEV and UC JEV were allowed to infect cells for 5, 10, 15, 20, 25, 30 and 60 min. Then the cells were imaged in 3D and the number of viruses inside the cells was determined with ImageJ (n = 50). (g) The membrane fusion efficiencies of viruses in cells infected by DiO-R18-labeled viruses (n = 50). (h) The efficiencies of the viruses’ different invasion stages when 2000 virus particles per cell were allowed to infect cells.

To specifically analyse the effect of ultracentrifugation and ultrafiltration purification on the virus invasion, JEV, ultrafiltered JEV, and ultracentrifuged JEV were incubated with Vero cells at 4°C at a concentration of 2000 particles per cell. It was found that the ultrafiltered JEV followed nearly the same attachment kinetics as the JEV, except that its attachment was slightly slower in the first 10 min (Figure 6a,b). As for the ultracentrifuged JEV, although it followed a similar attachment trend to the JEV, the amount of virus attached to the cell was significantly less than that of the JEV within 1 h of incubation (Figure 6a,b). Next, various numbers of viruses were incubated with the cells. It was found that as the number of viruses incubated with the cell increased from 500 to 30,000 particles per cell, the number of ultrafiltered JEV attached to the cell was first slightly less than that of the JEV, and then almost the same (Figure 6c,d). Meanwhile, unlike the JEV, which reached saturated attachment on the cell surface when the number of viruses incubated with the cell reached 10,000, the number of ultracentrifuged JEV attached to the cell continued to increase until the number of viruses incubated with the cell reached approximately 30,000 (Figure 6c,d). As the number of viruses incubated with the cell increased from 500 to 30,000 particles per cell, the number of ultracentrifuged JEV attached to the cell was significantly less than that of the JEV (Figure 6c). Specifically, when the number of viruses incubated with the cell was 2000 particles per cell, the numbers of JEV, ultrafiltered JEV, and ultracentrifuged JEV attached to the cell were approximately 257, 246, and 174, respectively (Figure 6c). The corresponding attachment efficiencies were approximately 13% (257/2000), 12% (246/2000), and 9% (174/2000).

After incubation with the cells for 20 min, the viruses attached to the cell surface were allowed to infect the cells at 37°C. By tracking virus infection in cells with EGFP-labelled clathrin, it was found that among the JEV particles attached to the cell surface, approximately 19% entered the cell through CME, about 14% recruited clathrin but failed to enter the cell, and the rest showed no obvious clathrin recruitment or entry (Figure 6e). The proportion of ultrafiltered and ultracentrifuged JEV particles entering the cell through CME and recruiting clathrin but failing to enter the cell was similar to that of JEV. This result suggests that ultrafiltration and ultracentrifugation purification had no obvious effect on the entry route of the virus. By statistically quantifying the virus in each cell, it was found that the number of ultrafiltered and ultracentrifuged JEV internalized by the cell increased as the infection time increased in the first 30 min, similar to the entry kinetics of JEV (Figure 6f). However, the number of the three types of viruses entering the cell was different. Roughly, when 2000 particles were incubated with the cell, about 53 JEV, 47 ultrafiltered JEV and 27 ultracentrifuged JEV particles entered the cell after infection for 30 min (Figure 6f). As shown in Figure 6c, the entry efficiencies of the viruses were approximately 21% (53/257), 19% (47/246), and 16% (27/174), respectively.

In addition, the membrane fusion of ultrafiltered and ultracentrifuged JEV was also analysed. After 3 h of infection, the fusion efficiencies of the ultrafiltered and ultracentrifuged JEV were both significantly lower than that of JEV (Figure 6g). As the infection time increased to 6 h, the fusion efficiency of the ultrafiltered JEV increased to the same level as that of the JEV (97%), whereas the fusion efficiency of the ultracentrifuged JEV was approximately 79%.

These results suggest that purifying viruses by ultracentrifugation affects virus attachment to the cell surface, entry into the cell, and fusion with the acidic endosome (Figure 6h). Ultrafiltration had no significant effect on the number of invasion steps.

## Discussion

In this study, we employed quantitative single-particle analysis to comprehensively analyse the invasion processes of SFV, JEV, and IAV under the same experimental conditions, including attachment to the cell surface, entry into the cell, transport towards the cell interior, and fusion with endosomes to release nucleocapsids.

Based on the above results, it could be concluded that the process of transport from the cell edge to the cell interior hardly limited viral invasion, but other steps might limit the invasion of different kinds of viruses to varying degrees (Figure 5e). Specifically, when the viruses infected the cells at low concentrations, the efficiencies of the SFV population to bind to the cell surface, enter the cell, and fuse with acidic endosomes were approximately 96%, 31%, and 97%, respectively. This is consistent with previous research, which demonstrated that over 80% of SFV added to BHK-21 cells could bind even at low temperatures, and 80% of internalized viruses released their nucleocapsids into the cytoplasm [22,30]. These data all demonstrate the high efficiency of SFV invasion. During JEV invasion, the efficiencies of the three invasion steps were approximately 27%, 10%, and 97%, respectively. During IAV invasion, the efficiencies of the three invasion steps were approximately 5%, 9%, and 53%, respectively. These results suggest that the process of entering cells is a severe invasion-limiting process for different types of viruses. For viruses with low PFU-to-particle ratios, the processes of attachment to the cell surface and even fusion with endosomes might also greatly limit virus invasion [31]. In conclusion, viruses with different IU-to-particle ratios have different limiting steps in the early stages of infection.

Additionally, our study revealed that the attachment process is a pivotal step in viral invasion and inefficient attachment significantly reduces virus infectivity. Interestingly, SFV, a highly infectious virus, exhibited remarkably high attachment efficiency (96%). The attachment efficiency of JEV was 27%, whereas IAV with low infectivity demonstrated a correspondingly low attachment efficiency (5%). The results showed that the size of cells or the number of associated receptors were not the reason for the variations in virus attachment efficiency. This indicates that differences in the binding force between viral glycoproteins and receptors or the quantity of glycoproteins with exposed receptor-binding sites may contribute to variations in virus attachment efficiency. The discrepancy in attachment efficiency has been identified as a crucial factor contributing to the differing infectivity levels among viruses.

Furthermore, our study revealed that the entry efficiencies of SFV, JEV, and IAV particles were different. SFV, JEV, and IAV enter host cells mainly via clathrin-mediated endocytosis (CME) [10,18,19]. According to our previous findings, the key reason for the low entry efficiencies of the viruses might be that most of the virus particles on cell surfaces were unable to recruit sufficient endocytosis-related proteins, such as clathrin and dynamin, to assist their entry [10]. SFV enters cells primarily through pre-existing clathrin-coated pits, whereas IAV enters cells by recruiting clathrin to form de novo clathrin-coated pits [10,32]. The differences in entry kinetics and efficiency between SFV, JEV, and IAV might be caused by the different targeting methods of clathrin-coated pits. It was also found that the fusion efficiency of SFV and JEV were similar, while the fusion efficiency of IAV was lower. According to previous reports, SFV and JEV fused with early endosomes at pH 6.5 ~ 6.0, while IAV fused with late endosomes at pH 6.0 ~ 5.0 [17,33]. The membrane fusion efficiencies of SFV and JEV were similar (Figure 5d). However, fusion of SFV occurred earlier than that of JEV (Figure 5c), implying that the endosomal pH triggering SFV fusion might be higher than that triggering JEV invasion. The low fusion efficiency of IAV might be caused by some virions lacking the ability to fuse with endosomes, and some virions failing to fuse with late endosomes in time and were degraded by lysosomes [34].

However, the complete infection cycle of viruses involves additional critical stages including uncoating, genome replication, protein synthesis, progeny virus assembly, and budding. The labelling of viral genomes or capsids typically requires genetic engineering of the viral genome to introduce the desired tags, and these methods often have suboptimal labelling efficiency, rendering them unsuitable for quantitative analysis [7]. Therefore, it is essential to quantitative analysis of uncoating and subsequent steps by developing new genome labelling strategies.

By analysing the invasion efficiency at different virus concentrations, we found that the attachment and entry efficiency were dependent on the number of viruses encountering the cell, whereas the transport and membrane fusion efficiency were not influenced by virus concentration. In detail, the more virus particles that were incubated with the cell, the lower the proportion of viruses attached to the cell surface. This indicates that when the number of viruses was limited, there were sufficient receptors available for binding. However, when the number of viruses increases, many cannot encounter receptors with available binding sites. The viruses may also be hindered by steric hindrance and charge repulsion from those already on the cell membrane, resulting in decreased efficiency with increasing virus concentration. Polycations can promote viral invasion by stabilizing virus attachment and preventing rapid dissociation from receptors [35]. In contrast to the attachment efficiency curve, the entry efficiency increased with an increase in the virus concentration, as depicted in Figure 3e. In other words, when a cell is infected by only a few viruses, the attachment process for the viruses may be relatively straightforward, whereas the entry process is more challenging. As more viruses infect a cell, attachment might have become more difficult, but virus entry becomes easier. As virus invasion varies with virus concentration, this result emphasizes the importance of paying attention to virus concentration in virus research, especially when discussing invasion efficiency.

Finally, we analysed the effect of ultracentrifugation and ultrafiltration purification on virus invasion and found that purifying virus particles through ultracentrifugation significantly impacted each key invasion stage, whereas ultrafiltration purification remained unaffected. These results indicate that the ultrafiltration may be the preferred choice for virus purification. In our study, using the lipid-specific method, we used the virus directly without ultracentrifugation or ultrafiltration purification, excluding the effect of purification on viral invasion.

In virus research, it is crucial not only to understand virus invasion but also to ascertain the efficiency of viruses at specific steps. By quantifying the virus invasion process and measuring the efficiency at each step, we can gain a more comprehensive understanding of virus invasion. In this work, we report the efficiencies of different invasion stages of three viruses with different infectivities in parallel for the first time, contributing to a deeper understanding of virus invasion and offering a protocol for the quantitative analysis of virus invasion efficiency.

## Supplementary Material

Supplemental Material

## Data Availability

The data supporting the findings of this study are openly available in the Science Data Bank at https://doi.org/10.57760/sciencedb.15988
