# Supplementary material for: Analyzing the factors affecting virus invasion by quantitative single-particle analysis
Source: Virulence. 2024 Jun 23;15(1):2367671. doi: 10.1080/21505594.2024.2367671 (PMC11197921; doi:10.1080/21505594.2024.2367671)
Supplement: Supplemental Material [file KVIR_A_2367671_SM0264.docx]

**Supporting Information**

**Analyzing the factors affecting** **the virus invasion by quantitative single-particle analysis**

Yi-Ning Hou^1, #^, Li-Juan Zhang^1, #^, Lei Du^1^, Dan-Dan Fu^1^, Jing Li^1^, Liu Liu^1^, Peng-Fei Xu^1^, Ya-Wen, Zheng^1^ Dai-Wen Pang^2,^ *, Hong-Wu Tang^1,^ *

^1^College of Chemistry and Molecular Sciences, Wuhan University, Wuhan 430072, P. R. China

^2^College of Chemistry, Nankai University, Tianjin 300071, P. R. China.

^#^Yi-Ning Hou and Li-Juan Zhang contributed equally to this work.

*All communications should be addressed to Hong-Wu Tang (hwtang@whu.edu.cn) and Dai-Wen Pang (dwpang@whu.edu.cn).


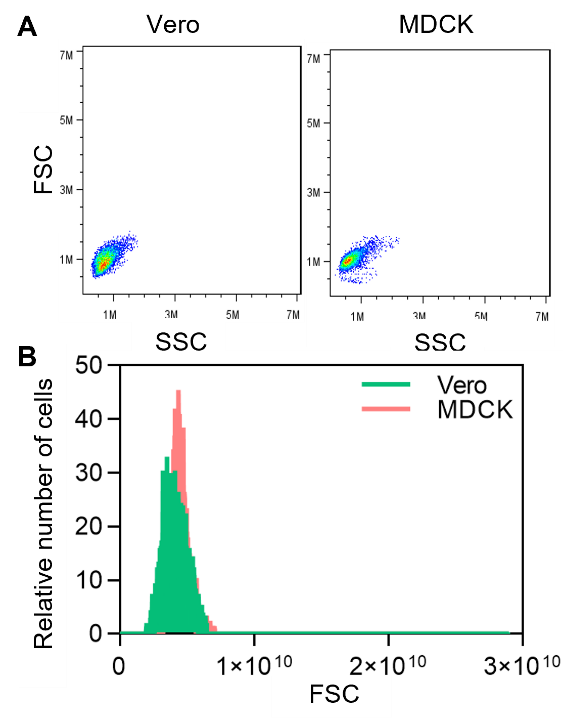


**Figure S1**. The sizes of Vero and MDCK cells. (a) FSC vs SSC and (b) FSC vs cell number (relative) of Vero and MDCK cells measured by flow cytometry.


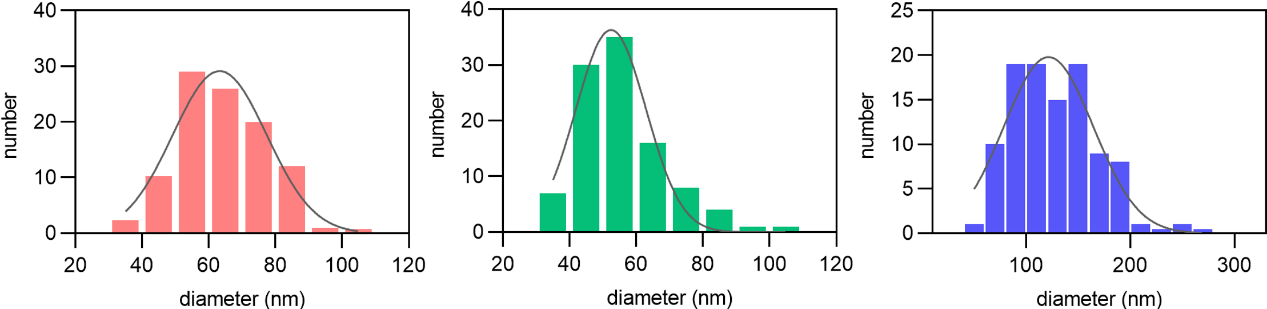


**Figure S2**. The size of SFV, JEV and IAV particles. Diameter vs number plot of SFV, JEV and IAV measured by transmission electron microscope (n = 100).


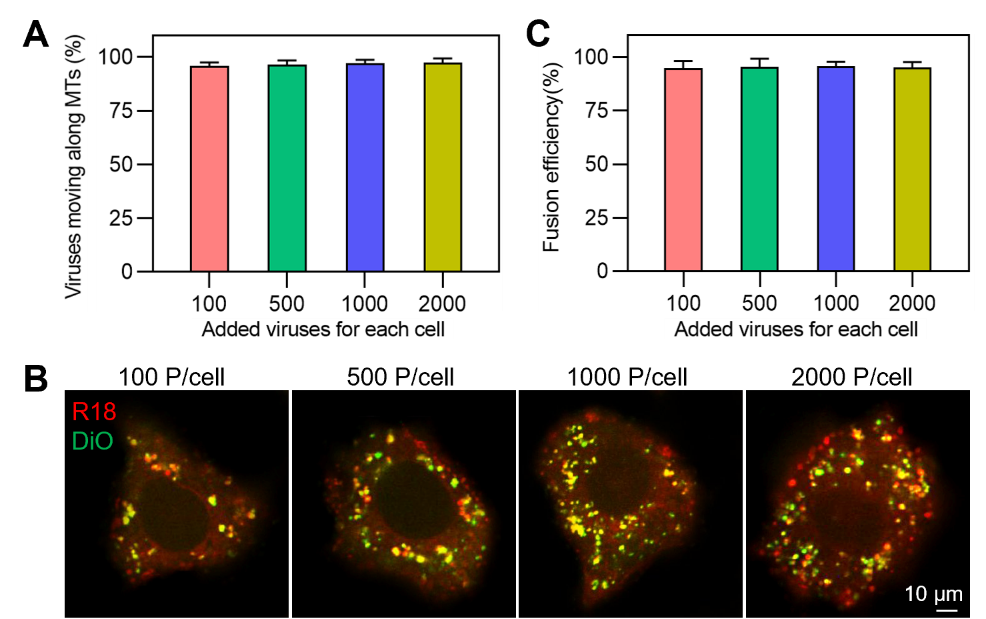


**Figure S3.** The transport efficiency and fusion efficiency of SFV when different numbers of virus particles were incubated with the cells. (a) The transport efficiencies of SFV. (b and c) The images and the quantified fusion efficiencies of SFV after infection for 3 h.
